# Relationship between Mediterranean diet and periodontal inflammation in a UK population: A cross‐sectional study

**DOI:** 10.1002/jper.70016

**Published:** 2025-09-15

**Authors:** Giuseppe Mainas, Giuseppe Grosso, Jason Di Giorgio, Joshua Hurley, Meaad Mohammed Alamri, Gaetano Isola, Mark Ide, Luigi Nibali

**Affiliations:** ^1^ Periodontology Unit Centre for Host Microbiome Interactions Faculty of Dentistry Oral & Craniofacial Sciences, King's College London London UK; ^2^ Department of Biomedical and Biotechnological Sciences University of Catania Catania Italy; ^3^ Dental Health Department College of Applied Medical Sciences King Saud University Riyadh KSA; ^4^ Department of General Surgery and Surgical–Medical Specialties Unit of Periodontology School of Dentistry University of Catania Catania Italy

**Keywords:** c‐reactive protein, diet, Mediterranean, inflammation, interleukins, matrix metalloproteinases, periodontitis, serum

## Abstract

**Background:**

Evidence is emerging about the effects of a balanced nutrition in maintaining periodontal health. The aim of this project was to investigate the association between diet, severity of periodontitis, and periodontal and systemic inflammation.

**Methods:**

Two hundred consecutive hospital patients underwent a full periodontal assessment, had blood samples taken, and filled out a food frequency questionnaire (FFQ). Adherence to a Mediterranean‐type diet was assessed through the FFQ. High‐sensitivity C‐reactive protein (hs‐CRP) serum levels of matrix metalloproteinases‐8 (MMP‐8), interleukin (IL)‐1α, IL‐1β, IL‐6, IL‐10, and IL‐17 were analyzed. Correlation and multivariate logistic regression analyses were performed to test the relationships between dietary factors, inflammatory biomarkers, and clinical data.

**Results:**

A total of 195 patients had complete data, with 112 participants categorized as highly adherent to the Mediterranean diet. Multivariate analysis showed that low adherence to Mediterranean diet was associated to periodontitis stage III–IV (*p *= 0.055, odds ratio [OR] 0.35, 95% confidence interval [CI]: 0.12–0.89); among individual food groups, more frequent red meat consumption was independently associated with more severe periodontitis stage (*p *= 0.042, OR 2.75, 95% CI: 1.03–7.41). Periodontal disease severity showed moderate associations with both circulating hs‐CRP and IL‐6 in the univariate analysis, but only IL‐6 association was confirmed after adjusting for confounders. Consumption of several plant‐derived food groups was significantly inversely related to increased levels of hs‐CRP, IL‐1α, IL‐6, IL‐10, and IL‐17.

**Conclusions:**

This study shows that low adherence to Mediterranean diet and higher red meat consumption may be associated with severity of periodontal disease. Studies with a larger sample size are needed to further clarify the current findings.

**Plain Language Summary:**

This study explored how everyday eating habits might impact gum health and overall inflammation. We evaluated 200 hospital patients by performing dental exams, taking blood samples, and asking them about their diets through questionnaires. In particular, we looked at how closely people followed a Mediterranean‐style diet, known for its emphasis on fruits, vegetables, whole grains, and healthy fats. Our findings revealed that patients who did not follow the Mediterranean diet as closely tended to have more severe gum disease, especially if they consumed red meat frequently. We also observed that higher levels of a key inflammatory marker, such as interleukin‐6 (IL‐6), were linked to worse gum health, while diets rich in plant‐based foods were associated with lower levels of various inflammatory markers. These results suggest that a balanced, Mediterranean‐type diet could be important in reducing gum disease and systemic inflammation. Further studies with larger groups are needed to confirm these promising observations.

## 1 INTRODUCTION

Periodontitis is a microbially driven, host‐mediated disease that leads to loss of periodontal attachment and bone, with tooth loss as ultimate sequela.^1^ Periodontal disease is characterized by local inflammation, which can influence systemic inflammation and possible correlation with other systemic diseases.^2^ At molecular level, the activation of the immune system due to the microbiological and physical insult leads to production of inflammatory biomarkers.^3^ Among them, C‐reactive protein (CRP) is generally overexpressed in patients with periodontal disease compared with periodontally healthy individuals.^4^ Several studies observed that CRP is a marker of association between periodontitis and systemic diseases, with inflammation as a common denominator.^5^, ^6^, ^7^, ^8^ Given the complex relation between periodontitis and several other noncommunicable diseases, up to date it is unclear whether the activation of the immune system is entirely driven by the periodontal disease, or rather worsened in the context of other comorbid conditions.^8^ In this scenario, studying factors (and specific interventions)^9^ influencing systemic inflammation is of great interest to understand if they could possibly affect disease severity.

Recent studies have reported that an unbalanced diet with consumption of processed/ultraprocessed and pro‐inflammatory foods might lead to an impairment of periodontal conditions,^10^ compared with consumption of a diet rich in salad, fruit, and vegetables, which was found to improve the periodontal condition.^11^, ^12^ Among the most studied dietary patterns, the Mediterranean diet is a plant‐based diet and one of the most studied dietary patterns and renowned as a healthy approach worldwide.^13^ Although over the years, Mediterranean diet has been enriched by other foods in different populations, it mainly consists of a high consumption of fruits, vegetables, legumes, whole‐grain cereals, and nuts, with olive oil as the main source of lipids, a moderate consumption of animal products, such as dairy, fish, eggs, and meat (ideally unprocessed), moderate consumption of wine (especially red, during meals), and limited sweets and processed food products.^14^ According to the existing literature, higher adherence to the Mediterranean diet has been associated with lower risk of developing major noncommunicable diseases, including cardiovascular diseases, neurodegenerative disorders, and certain cancers.^15^, ^16^ Furthermore, a study found that a lifestyle characterized by a low adherence to the Mediterranean diet led to a sizeable risk of having periodontitis,^17^ which was confirmed by a very recent systematic review.^18^

To date, a very large study investigated the effects of dietary habits on the risk of periodontal disease in a UK population;^19^ however, detailed periodontal examination and the relation with biomarkers of inflammation have not been explored so far. Therefore, the aim of this study was to investigate the associations between diet (particularly adherence to a Mediterranean‐type diet), periodontal disease severity, and systemic inflammation.

## 2 MATERIALS AND METHODS

This was a cross‐sectional study. The Strengthening the Reporting of Observational Studies in Epidemiology (STROBE) guidelines were followed, available in Supporting Material 1 in the online *Journal of Periodontology*.

**Research question**: Do dietary habits affect periodontal disease and its associated systemic inflammatory response?

### 2.1 Study population

Samples were collected from consecutive patients taking part in the King's College London Oral, Dental, and Craniofacial Biobank and providing dietary data and blood samples. The Biobank was granted ethics approval by the East of England–Cambridge East Research Ethics Committee (reference 20/EE/0241). The study was conducted according to the principles of the Declaration of Helsinki. The privacy rights of human subjects have been observed, and each patient gave written consent to take part in the Biobank recruitment in the Periodontology new patients’ clinic at the Guy's & St Thomas Foundation Trust Hospital (GSTFT). Specific approval for the release of data and samples for this study was granted by the Biobank Management Committee (Biobank reference 020). Two hundred consecutive patients were recruited between April 2023 and May 2024.

#### 2.1.1 Inclusion criteria

1. Individuals who consented to participate in the King's College London Oral, Dental, and Craniofacial Biobank
2. Individuals who completed full clinical periodontal assessments and periodontal diagnosis available
3. Individuals who accepted to donate serum samples
4. Individuals who accepted to complete the food frequency questionnaire (FFQ).

#### 2.1.2 Exclusion criteria

1. Periodontal treatment (especially if in a specialist setting) received within the previous 12 months
2. Unreliable or incomplete FFQ data as assessed by internal data quality checks.

### 2.2 Clinical examination

Following consent, demographic and medical parameters were collected, along with dental and medical history. Self‐reported smoking habit was recorded (number of cigarettes/days, years of smoking). Height, weight, and waist measurements were taken at the study visit. Periodontal examinations were performed by several clinicians at the point‐of‐care setting, as part of routine diagnostic assessments within the Biobank protocol. The following periodontal measurements were taken at 6 sites/tooth by Biobank examiners using a UNC‐15 periodontal probe: dichotomous full mouth plaque scores (FMPS), full mouth probing pocket depth (PPD), recession (REC) of the gingival margin from the cemento‐enamel junction (CEJ), bleeding on probing (BOP), tooth mobility, and furcation involvement. Clinical attachment level (CAL) was calculated as PPD+REC.

### 2.3 Periodontal diagnosis

Periodontal diagnosis was based on the current classification of periodontal diseases,^20^ using the following criteria:

1. Periodontal health: BOP <10% and PPD ≤4 mm and no site ≥4 mm PPD with BOP with evidence of previous bone loss/attachment loss
2. Gingivitis: BOP ≥10% with all sites with PPD ≤3 mm
3. Periodontitis: presence of ≥2 nonadjacent sites with PD 4 mm and BOP or >4 mm PPD with evidence of radiographic bone loss (except in case of deep caries, endo‐periodontal pathology, fracture, third molars).^21^ Patients with periodontitis were further subdivided according to staging and grading.^20^

### 2.4 Serum collection

Blood samples were collected from each participant using standard venipuncture techniques. Blood samples consisted of 2 tubes: 1 for whole blood and 1 for serum. A total of 13–15 mL of whole blood was drawn into sterile vacutainer tubes containing clot activator and gel separator. The tubes were gently inverted to ensure proper mixing of the anticoagulant and the blood sample. Blood was immediately aliquoted in Eppendorfs and stored at −80°C. Subsequently, the other samples were allowed to clot at room temperature for 30 min to 1 hour to facilitate serum separation. Following clot formation, the tubes were centrifuged at 4000 rpm for 5 min. This centrifugation step effectively separated the serum from the clot and cellular components. The resulting serum samples were then carefully transferred into labelled microcentrifuge tubes using a sterile pipette and stored at −80°C until analysis at King's College London.

### 2.5 Laboratory analysis

Enzyme‐linked immunosorbent assay (ELISA) was performed in order to detect circulating levels of highly‐sensitive C‐reactive protein (hs‐CRP). High‐sensitivity Multi‐Analyte ELISAs (Ella Automated Immonoassay System; ProteinSimple) was performed in order to assess serum levels of IL‐1α, IL‐1β, IL‐6, IL‐10, IL‐17, and MMP‐8, according to manufacturer instructions. Each sample was analyzed in duplicate for both procedures (more details can be found in Supporting Material 2 in the online *Journal of Periodontology*).

### 2.6 Dietary habits

A 37‐item food‐frequency questionnaire (FFQ) referring to the dietary habits of the previous 6 months was self‐administered to each patient to record their weekly food consumption. The FFQ was inspired by a tool previously validated in the clinical setting^22^ with the inclusion of specific subgroups of fruit (i.e., berries, citrus fruits), vegetables (i.e., green leafy vegetables), legumes (i.e., beans) in addition to the general food groups. Adherence to the Mediterranean diet was assessed through the application of a validated Mediterranean diet adherence score (MedDietScore) elaborated from previous studies and widely used in the scientific literature.^23^ For its calculation, the weekly consumption of 9 food groups in line with the principles of the Mediterranean diet (fruit, vegetables, nonrefined cereals, legumes, fish, meat, poultry, dairy, olive oil, and alcohol) was assessed to assign the relative rating (from 0 to 5, or the reverse) in each of the food groups according to their position in the Mediterranean diet pyramid. Specifically, plant‐based foods followed a monotonic crescent function while meat products, poultry, and dairy consumption was assigned a reversed scoring scale. Especially for alcohol, the optimal intake considered within the paradigm of the Mediterranean diet is moderate, hence a score of 5 was assigned for consumption of less than 300 mL/day, score of 0 for no consumption of >700 mL/day and scores 4 to 1 for decrescent consumptions from 700 to 300 mL/day. The total score ranges from 0 to 55, with higher values indicating higher adherence to the Mediterranean diet. For the purposes of this study, the median score was used as a cutoff point to categorize low and high adherence to the Mediterranean diet.

### 2.7 Statistical analysis

This study utilized a convenience sample of 200 consecutive patients based on the simultaneous availability of both a completed FFQ and stored blood samples for biomarker analysis. No formal a priori sample size calculation was conducted, as this was an exploratory, cross‐sectional investigation designed to assess associations between dietary patterns, periodontal status, and systemic inflammation within the limits of available data.

The main hypothesis to be tested was whether dietary habits might modulate the associations between severity of periodontal disease and circulating CRP levels. The study primary outcome was hs‐CRP serum levels. Secondary outcomes were serum levels of MMP‐8, IL‐6, IL‐1α, IL‐1β, IL‐10, and IL‐17. The main explanatory variables used in the analyses were periodontal diagnosis, dietary patterns, and individual food consumption. Associations between dietary data (particularly adherence to the Mediterranean diet dichotomized as high versus low based on the median MedDietScore cutoff value) and periodontal phenotype were also sought.

Categorical variables are presented as absolute numbers and relative frequencies (%), continuous variables are reported as means and standard deviations (SDs). Differences between groups were assessed by Chi‐square test for categorical variables and ANOVA or Kruskal–Wallis test for variables normally and not normally distributed, respectively. Pearson's correlation coefficients were calculated to assess the correlation between biomarkers of inflammation and individual food group intake. Logistic regression analyses were conducted to calculate the odds ratios (ORs) and 95% confidence intervals (CIs) for the association between adherence to the Mediterranean diet, individual food group intake, and severity of disease. Multivariate analyses were performed to rule out possible confounding factors known to be associated with the periodontitis (including age, sex, ethnicity, index of multiple deprivation (IMD), smoking history, frequency of toothbrushing/day, interdental cleaning, and body mass index [BMI]). All statistical analyses were performed using SPSS (SPSS Inc., Chicago, IL, USA) software Version 29.

## 3 RESULTS

The main characteristics of patients are shown in Table 1. Following exclusion of 5 dietary questionnaires with unclear/unreliable data, a total of 195 FFQs were analyzed. Most of the included patients were never smokers (55.7%), followed by former smokers (34.4%), and current smokers (9.4%). Most participants (*n* = 170) were diagnosed with stage III–IV periodontitis, while 16 had gingivitis and 14 were periodontally healthy. Eighty‐three participants were categorized to have low adherence to Mediterranean diet, whereas 112 showed high adherence to Mediterranean diet. Individuals with high adherence to the Mediterranean diet were predominantly never‐smokers compared to those with low adherence. Additionally, subjects with high adherence to Mediterranean diet exhibited a lower grade of periodontitis, lower mean probing pocket depth, reduced clinical attachment loss, and fewer periodontal pockets exceeding 4 and 5 mm in depth compared to the low‐adherence group.

**TABLE 1:** Background characteristics and clinical parameters of the study sample by level of adherence to the Mediterranean diet (*n* = 195).

		Mediterranean diet adherence		
Parameter	Total (*n* = 195)	Low (*n* = 83)	High (*n* = 112)	*p*‐value
Sex, *n* (%)				0.159
Male	66 (34.0)	32 (38.6)	34 (30.6)	
Female	128 (66.0)	51 (61.4)	77 (69.4)	
Age (y), mean (SD)	48.8 (13.9)	48.1 (13.7)	49.3 (14.1)	0.554
Ethnicity				0.240
White Caucasian	106 (57.6)	46 (57.5)	60 (57.7)	
Black African/Caribbean	38 (20.7)	21 (26.3)	17 (16.3)	
Asian	29 (15.8)	9 (11.3)	20 (19.2)	
Any other/mixed	11 (6.0)	4 (5.0)	7 (6.7)	
Smoking status, *n* (%)				0.003
Never	107 (55.7)	35 (42.7)	72 (65.5)	
Former	66 (34.4)	34 (41.5)	32 (29.1)	
Current	18 (9.4)	13 (15.9)	5 (4.5)	
BMI, mean (SD)	28.28 (16.1)	28.55 (11.8)	28.06 (18.8)	0.840
IMD, *n* (%)				0.716
1 (most deprived areas)	39 (20.0)	16 (41.0)	23 (59.0)	
2	39 (20.0)	16 (41.0)	23 (59.0)	
3	40 (20.5)	21 (52.5)	19 (47.5)	
4	38 (19.5)	15 (39.5)	23 (60.5)	
5 (least deprived areas)	39 (20.0)	15 (38.5)	24 (61.5)	
Health status, *n* (%)				
Type‐2 diabetes	21 (11)	10 (12.2)	11 (10.1)	0.408
CVD	5 (2.6)	2 (2.4)	3 (2.8)	0.632
Hypertension	36 (18.8)	15 (18.3)	21 (19.3)	0.509
Rheumatic disease	12 (6.3)	6 (5.5)	6 (7.4)	0.404
Others	88 (46.6)	39 (48.1)	49 (45.4)	0.408
Periodontitis stage, *n* (%)				0.022
0–2	40 (20.5)	11 (13.3)	29 (25.9)	
3–4	155 (79.5)	72 (86.7)	83 (74.1)	
Periodontitis grade, *n* (%)				<0.001
0	28 (14.4)	7 (8.4)	21 (18.8)	
A	4 (2.1)	3 (3.6)	1 (0.9)	
B	31 (15.9)	5 (6.0)	26 (23.2)	
C	132 (67.7)	68 (81.9)	64 (57.1)	
Periodontitis extent, *n* (%)				0.155
No	28 (14.4)	9 (10.8)	19 (17.0)	
Localized	53 (27.2)	19 (22.9)	34 (30.4)	
Generalized	114 (58.5)	55 (66.3)	59 (52.7)	
PPD, mean (SD) (mm)	3.03 (0.96)	3.29 (0.92)	2.83 (0.96)	0.002
REC, mean (SD) (mm)	0.75 (0.75)	0.76 (0.77)	0.74 (0.74)	0.850
CAL, mean (SD) (mm)	3.75 (1.35)	4.02 (1.29)	3.56 (1.36)	0.032
No. PPDs > 4 mm, mean (SD)	24.33 (24.30)	30.69 (26.19)	19.58 (21.73)	0.002
No. PPDs > 5 mm, mean (SD)	8.49 (12.3)	10.82 (12.5)	6.75 (11.99)	0.028

*Note*: *p* < 0.05.

Abbreviations: BMI, body mass index; CVD, cardiovascular diseases; CAL, clinical attachment level; IMD, index of multiple deprivation; PPD, probing pocket depth; REC, recession; SD, standard deviation.

Table 2 shows results of analyses comparing circulating hs‐CRP levels across different periodontal phenotypes. No statistically significant differences were detected when analyzing periodontal health versus gingivitis versus periodontitis (*p* = 0.765) and when analyzing differences by staging (*p* = 0.120), while there was an association when grouping patients by no‐periodontitis (healthy and gingivitis) versus mild–moderate periodontitis (stage I and II) and severe periodontitis (stage III and IV) (*p* = 0.036). When other serum markers were investigated (Table 2), associations were detected for IL‐6 levels by staging (*p *= 0.019) and by no versus mild–moderate versus severe periodontitis (*p* = 0.042). Multivariate analysis (adjusted for Mediterranean diet score, age, sex, ethnicity, IMD, smoking history, diabetes type II, CVD, hypertension, rheumatic disease, other diseases, frequency of toothbrushing/day, interdental cleaning, and BMI) revealed that hs‐CRP lost its association with periodontal stages (*p* = 0.272), whereas IL‐6 kept it for both grouping by stages (*p* = 0.014) and by no versus mild‐moderate versus severe periodontitis (*p* < 0.001) (see Supporting Material 3 in the online *Journal of Periodontology*).

**TABLE 2:** Median and IQR values divided by diagnosis.

Parameter	hs‐CRP (mg/L)	IL‐1β (pg/mL)	IL‐1α (pg/mL)	IL‐6 (pg/mL)	IL‐10 (pg/mL)	IL‐17 (pg/mL)	MMP‐8 (pg/mL)
Healthy (n=14)	1.22 (0.51–1.47)	0.003 (0.003–0.017)	0.001 (0.001—0.037)	1.53 (1.04–2.76)	1.13 (0.82–1.55)	0.38 (0.01–1.11)	67611.00 (30389.00–221348.50)
Gingivitis (n=16)	1.63 (1.34–2.27)	0.003 (0.003–0.003)	0.001 (0.01–0.07)	2.62 (1.64–6.07)	1.25 (0.99–1.77)	0.18 (0.01–0.61)	78320.00 (49303.00–102893.50)
Periodontitis stage I (n=2)	2.41 (2.31–2.51)	0.003 (0.003–0.003)	0.53 (0.001–0.105)	3.35 (2.74–3.97)	1.91 (1.88–1.94)	0.27 (0.01–0.53)	84403.50 (75209.00–93598.00)
Periodontitis stage II (n=11)	1.47 (1.34–1.93)	0.003 (0.003–0.003)	0.001 (0.001–0.001)	2.50 (1.64–5.47)	1.13 (0.99–1.34)	0.18 (0.01–0.61)	78320.00 (49303.00–99106.50)
Periodontitis stage III (n=99)	1.08 (0.50–1.62)	0.003 (0.003–0.003	0.01 (0.001–0.0027)	1.96 (1.48–3.18)	1.15 (0.92–1.48)	0.13 (0.01–0.36)	68382.00 (33655.50–139438.50)
Periodontitis stage IV (n=58)	1.28 (0.49–2.02)	0.003 (0.003–0.004)	0.01 (0.001–0.001)	2.35 (1.84–3.80)	1.30 (0.94–1.56)	0.15 (0.01–0.61)	55983.00 (29791.00–127551.00)
p‐Values for differences across groups ^a^	0.036	0.164	0.573	0.042	0.624	0.173	0.335
p‐Values for differences across stages ^b^	0.120	0.403	0.444	0.019	0.209	0.472	0.445

Abbreviations: hs‐CRP, high‐sensitivity C‐reactive protein; IL, interleukins; IQR, interquartile range; MMP, matrix metalloproteinases.

^a^ Groups were divided by 0 (no periodontitis + gingivitis), 1 (stages I‐II), and 2 (stages III–IV).

^b^ Stages were divided by 0 (no periodontitis + gingivitis) and I, II, III, and IV.

Low adherence to Mediterranean diet was statistically significantly correlated to periodontitis stage III–IV, as revealed by the multivariate analysis (adjusted for age, sex, ethnicity, IMD, smoking history, diabetes type II, CVD, hypertension, rheumatic disease, other diseases, frequency of toothbrushing/day, interdental cleaning, and BMI) (OR 0.35, 95% CI 0.12–0.89) that is shown in Table 3.

**TABLE 3:** Association between adherence to the Mediterranean diet and severe periodontitis (stage III–IV).

Parameter	Mediterranean diet adherence, OR (95% CI)
	High vs. low
Unadjusted	0.43 (0.20–0.93)
Age‐, ethnicity‐ and sex‐adjusted	0.36 (0.15–0.84)
Multivariate^*^	0.35 (0.12–0.89)

Abbreviations: BMI, body mass index; CI, confidence interval; CVD, cardiovascular diseases; IMD, index of multiple deprivation; OR, odds ratio.

* Multivariate model adjusted for age, sex, ethnicity, IMD, smoking history, diabetes II, CVD, hypertension, rheumatic disease, other medical history, frequency of toothbrushing/day, interdental cleaning, and BMI.

Among food consumption, frequency of red meat and derived products intake was the only food category that was significantly linked to periodontitis stage III–IV, as observed in the multivariate analysis (OR 2.75, 95% CI 1.03–7.41) (Table 4).

**TABLE 4:** Association between major food group consumption and severe periodontitis (stage III–IV).

Parameter	OR (95% CI)^*^
	High vs. low consumption
Fruit	0.34 (0.10–1.07)
Vegetables	1.09 (0.35–3.41)
Legumes	0.92 (0.29–2.89)
Cereals	0.81 (0.29–2.19)
Fish	1.21 (0.45–3.27)
Red meat and derived products	2.75 (1.03–7.41)
Dairy	1.25 (0.42–3.72)
Olive oil	1.17 (0.34–3.99)

Abbreviations: BMI: body mass index; CI: confidence interval; IMD: index of multiple deprivation; OR: odds ratio.

* Multivariate model adjusted for age, sex, ethnicity, IMD, smoking history, frequency of toothbrushing/day, interdental cleaning, and BMI.

No statistically significant association was found between adherence to Mediterranean diet and all the assessed biomarkers (see Supporting Material 4 in the online *Journal of Periodontology*).

A significant inverse correlation was observed between frequency of vegetables, legumes, and dairy products consumption and hs‐CRP, with similar trends that were observed for other plant‐derived food consumption related to the other assessed biomarkers (Table 5).

**TABLE 5:** Correlation matrix between major food group consumption and biochemical markers of inflammation.

Parameter	Fruit	Vegetables	Legumes	Cereals	Fish	Meat	Dairy	Olive oil
CRP	−0.048	−0.156^*^	−0.146^*^	−0.095	−0.049	−0.006	−0.174^*^	−0.014
IL‐1β	−0.116	−0.051	0.066	0.092	0.027	−0.019	0.003	−0.038
IL‐1α	−0.141^*^	−0.015	−0.034	0.025	−0.120	0.007	0.122	−0.009
IL‐6	−0.086	−0.197^**^	−0.147^*^	−0.107	−0.017	−0.011	−0.046	−0.123
IL‐10	−0.203^**^	−0.210^**^	−0.021	−0.036	−0.107	−0.139	0.063	−0.110
IL‐17	−0.113	−0.227^**^	−0.100	−0.066	0.015	−0.090	0.061	−0.153^*^
MMP‐8	−0.105	−0.162^*^	0.026	−0.020	−0.056	0.030	−0.054	−0.095

*Note*: Numbers indicate Spearman correlation coefficients.

Abbreviations: hs‐CRP, high‐sensitivity C‐reactive protein; IL, interleukins; MMP, matrix metalloproteinases.

* *p* < 0.05.

** *p* < 0.001.

## 4 DISCUSSION

This cross‐sectional study investigated the association between adherence to a Mediterranean‐type diet, periodontitis severity, and systemic inflammatory markers, aiming to generate hypotheses regarding potential relationships. The most interesting finding was that adherence to Mediterranean diet was associated with healthier periodontal conditions after adjusting for confounders.

The pathogenesis of periodontitis involves complex interactions between microbial biofilms and the host immune response, which results in the release of various inflammatory mediators and proteolytic enzymes.^8^, ^24^ We hereby assessed several of the inflammatory mediators which might play significant roles in periodontal disease severity and in its systemic impact.

CRP is widely recognized as a biomarker of systemic inflammation.^25^, ^26^ Numerous studies have explored the relationship between periodontitis and CRP levels using a variety of research designs, including cross‐sectional, case‐control, and longitudinal approaches.^27^ Interest in the connection between CRP and periodontitis stems from evidence linking periodontal disease to cardiovascular disease (CVD). Studies indicate associations between periodontal conditions and heightened risks of myocardial infarction, stroke, and the underlying pathology of atherosclerosis.^28^, ^29^ Although these associations do not establish causation, elevated CRP levels in periodontitis are thought to partly explain this relationship. The inflammatory burden from periodontal disease may contribute to systemic inflammation, potentially amplifying cardiovascular risks.^30^ In the current study, no association between serum hs‐CRP levels and periodontitis was found, in contrast with the existing literature.^7^, ^8^ The lack of association may be due to the low number of individuals recruited and the features of the population assessed, which may have underestimated the effect of the periodontal disease.

The present study also showed an association between periodontal status and IL‐6 systemic levels, although no clear gradient effect by staging was observed. IL‐6 is upregulated during periodontitis and is implicated in both local and systemic inflammatory responses.^31^ Systemic IL‐6 levels are often elevated in patients with periodontitis and have been associated with systemic conditions such as cardiovascular disease and diabetes.^32^ Authors found that serum levels of IL‐6 were increased in patients with periodontitis compared to periodontally healthy individuals, and it may contribute to osteoclastogenesis and alveolar bone resorption, underscoring its dual role in promoting tissue destruction and systemic inflammation.^32^, ^33^

Multivariate analysis showed that low adherence to Mediterranean diet was significantly associated with more severe periodontitis (stages III–IV). These findings are in line with a recent study that reported how a lifestyle characterized by a low adherence to Mediterranean diet was associated to a risk of having periodontitis 9 times higher than a high adherence.^17^ Another recent study observed that Mediterranean diet adherence score was inversely associated with the severity of periodontitis among US individuals,^34^ and while a systematic review supported these findings,^18^ another systematic review failed to demonstrate a significant association.^35^

Among specific food groups included in the context of a Mediterranean‐type diet, more frequent meat consumption was the only one significantly associated to stage III–IV periodontitis, in agreement with a study showing that higher red meat consumption was associated with higher probing depth and bleeding on probing.^36^ Although this hypothesis has not been fully clarified yet, a possible explanation is that the heme iron deriving from red meat can be a catalyst for oxidative stress, which in turn could be an initiating step to chronic diseases.^37^ The conditions of a protein‐rich and slightly alkaline environment favor the proliferation of periodontal pathogens such as *P. gingivalis*.^38^, ^39^ More specifically, during active periodontitis, in subgingival environments with deep pockets and increased amount of gingival crevicular fluid (GCF), great protein availability and pH changes promote the growth of pathogenic bacteria involved in proteins degradation that are associated with periodontal disease progression.^39^, ^40^ A cross‐sectional study assessed the periodontal condition of 100 vegetarian patients compared with the periodontal status of 100 non‐vegetarian individuals and authors observed that the vegetarians had significantly less probing pocket depth, less bleeding on probing, less missing teeth, and better oral hygiene when compared with the non‐vegetarians.^41^ Another cross‐sectional study on 13,920 Hispanic/Latinos living in the United States reported that higher consumption of whole grains and fruits, and lower consumption of red/processed meats were associated with lower odds of severe periodontitis, after assessing their diet quality by using the Alternative Healthy Eating Index (AHEI‐2010).^42^ A systematic review confirmed the association between high saturated fat and protein intake with systemic inflammation and periodontal disease progression.^43^ A more recent project involving 651 patients related to the Oral Infections, Glucose Intolerance, and Insulin Resistance Study (ORIGINS) reported that dietary approaches rich in fruits, vegetables, whole grains, and other nutritionally rich plant foods were associated with lower oral microbial diversity and favorable ratios of pathogenic to commensal microbiota.^44^

However, no associations were detected between adherence to Mediterranean diet and the studied serum inflammatory biomarkers. In contrast with our results, a systematic review and meta‐analysis of randomized controlled trials (RCT) found that Mediterranean‐type diet led to a pronounced reduction of several inflammatory biomarkers including CRP, IL‐6, IL‐1β, IL‐8, and tumor necrosis factor‐alpha (TNF‐α), with less significant influence of DASH (Dietary Approaches to Stop Hypertension) and vegetarian/vegan diets.^45^ Another systematic review of observational studies reported that following Mediterranean diet together with other (not specified) anti‐inflammatory diet scores consistently decreased CRP and IL‐6 serum levels.^46^

Interestingly, a significant inverse correlation was observed between frequency of consumption of a diverse range of plants‐derived products (such as legumes, vegetables, fruits, and olive oil) and the majority of the analyzed biomarkers. There is substantial evidence showing that diet might play a role on human health by affecting the immune system and modulating systemic inflammation depending on its composition and proportion of macronutrients, micronutrients and phytochemicals.^47^ These findings complement the literature suggesting that bioactive compounds in plant‐based foods, such as polyphenols, fibers, and unsaturated fatty acids, modulate inflammatory responses by downregulating pro‐inflammatory cytokines and enhancing the production of anti‐inflammatory mediators.^48^, ^49^ This association underscores the potential role of a nutrient‐dense, plant‐rich diet in mitigating systemic inflammation and its implications for periodontitis. In addition, our findings are supported by other studies that reported stronger associations between individual food categories and specific cytokines.^50^ Nevertheless, taking into account the differences in dietary habits, population characteristics and methodological approaches, further longitudinal studies are warranted to confirm causality and explore mechanistic pathways underlying these interactions.

According to the existing literature, adherence to Mediterranean‐type dietary patterns is generally moderate to low across much of Europe, particularly outside the Mediterranean basin.^51^, ^52^ A large pan‐European survey of adults over 50 (including Croatia, Greece, Italy, Slovenia, and other EU states) found only about 37% of MD adopters rated their health “very good” to “excellent,” underscoring modest adherence in northern and eastern regions.^53^ Similarly, authors reported medium‐to‐low adherence across 5 Mediterranean and North African countries, with the lowest scores in Slovenia and highest in Italy, highlighting significant regional variability.^54^ In the United Kingdom specifically, Tong et al. found fewer than 1 in 5 adults had high Mediterranean Diet Scores in the Fenland Study, with adherence strongly linked to higher socioeconomic position.^55^ Most recently, Shannon and coworkers, using UK Biobank data, confirmed that high adherence to MEDAS and PYRAMID scores was uncommon, though those who did adhere demonstrated a 14%–23% lower risk of dementia.^56^ Overall, these findings demonstrated that Mediterranean‐type diets remain under‐adopted in the UK and parts of Europe.

An important remark has to be done concerning the imbalance in smoking status across dietary adherence groups, despite we adjusted for smoking in all multivariate analyses. Participants with high adherence to a Mediterranean‐style diet were significantly more likely to be never‐smokers, while current smokers were more frequent in the low‐adherence group. Authors observed that an inflammatory dietary pattern was significantly associated with higher risk of periodontitis only among non‐smokers, suggesting that the effect of diet may be attenuated or obscured in smokers due to their already elevated baseline inflammatory status.^57^ Similarly, another study found that adherence to anti‐inflammatory dietary patterns was associated with lower odds of periodontitis, but only after adjusting for smoking, underscoring the independent and potentially interacting effects of these exposures.^58^ On a mechanistic level, Dietrich et al. discussed how smoking induces chronic low‐grade inflammation through increased oxidative stress and altered cytokine profiles, pathways also influenced by diet,^59^ supporting the plausibility of synergistic or antagonistic interactions.^60^ Together, these findings indicate that smoking may not only act as a confounder but could potentially modify the association between diet and periodontal disease.

The main strength of this project is the comprehensive investigation of a possible association between diet, systemic inflammation, and periodontitis. This investigation might be a starting point that can lead to more complex and specific future analyses to better understand the relationships between foods intake and periodontitis and to develop personalized management approaches. In addition, this study included the analysis of 7 biomarkers, 6 of which were assessed using the highly sensitive and innovative ELLA platform, a multiplex immunoassay technique comparable to a multiple ELISA.

However, the study has also some limitations to be addressed. First, the cross‐sectional design of the study does not allow to assess causality but only associations. Moreover, the timing of the relations retrieved is unmeasured, hence we are not able to discern the specific contribution of the disease and dietary habits to the systematic inflammation and vice versa. Second, the study included a relatively small sample size, which may suffer from statistical underpower when adjusting for several variables. Third, although the use of FFQ is a gold standard for such a type of study, the tools involved may still be limited to estimate exact portion sizes; in addition, the reporting may also suffer from recall and social desirability biases. Fourth, the imbalance in smoking status between diet adherence groups, despite statistical adjustment, may have introduced residual confounding, given the strong and independent association between smoking and periodontal disease severity.

## 5 CONCLUSIONS

In conclusion, our findings highlight that low adherence to Mediterranean diet and higher red meat consumption may be associated to severe periodontal disease. Since some associations were detected between periodontal disease severity, dietary factors, and biomarkers of inflammation, these aspects should be holistically considered when assessing periodontitis and inflammation. These results have to be interpreted with caution, and studies with larger sample size and more detailed food frequency questionnaires and details about caloric consumption should be carried out.

## AUTHOR CONTRIBUTIONS

Giuseppe Mainas contributed to conception, design, data acquisition, and interpretation and drafted the manuscript. Mark Ide contributed to conception, design, interpretation, and drafted the manuscript. Giuseppe Grosso and Jason Di Giorgio contributed to conception, data analysis, interpretation, and critically revised the manuscript. Joshua Hurley, Gaetano Isola, and Meaad Alamri contributed to data acquisition and critically revised the manuscript. Luigi Nibali contributed to interpretation, design, data acquisition, and drafted and critically revised the manuscript. All authors reviewed the final version and agreed to be accountable for all aspects of the work.

## CONFLICT OF INTEREST STATEMENT

The authors declare no conflicts of interest.

## Supporting information



Supporting Information

Supporting Information

Supporting Information

Supporting Information

## Data Availability

The data that support the findings of this study are available from the corresponding author upon reasonable request.
