# Supplementary material for: Relationship between Mediterranean diet and periodontal inflammation in a UK population: A cross‐sectional study
Source: J Periodontol. 2025 Sep 15;97(1):85–96. doi: 10.1002/jper.70016 (PMC12902710; doi:10.1002/jper.70016)
Supplement: Supplementary file 2 — Supporting Information [file JPER-97-85-s001.docx]

**Supplementary material 2**

**ELISA procedure.**

The enzyme-linked immunosorbent assay (ELISA) was performed using the **Instant Human Recombinant Kit** from Thermo Fisher Scientific to quantify serum inflammatory biomarkers. This kit uses a sandwich ELISA format, optimized for high sensitivity and rapid processing. Serum samples were thawed, gently mixed, and diluted according to the manufacturer’s recommendations to ensure the biomarker concentrations were within the standard curve range. The pre-coated 96-well plates were loaded with the diluted samples, standards, and controls in duplicate. After an incubation period, the plates were washed to remove any unbound material using a multi-step wash protocol. Detection antibodies specific to the target proteins were then added, followed by the addition of the horseradish peroxidase (HRP)-conjugated secondary antibody. The reaction was visualized by adding the **tetramethylbenzidine (TMB) substrate**, which produces a colour change proportional to the analyte concentration. The reaction was stopped using **sulfuric acid**, and absorbance was measured at **450 nm** using a microplate reader. The concentrations of biomarkers were calculated based on standard curves generated from recombinant proteins provided in the kit. The kit's built-in quality controls and validation steps ensured the accuracy, reproducibility, and reliability of the results across all serum samples analysed.

**ELLA procedure.**

The ELLA platform (Ella™ Automated Immonoassay System; ProteinSimple) was used to perform multiplex analysis of serum inflammatory biomarkers, allowing simultaneous quantification of multiple analytes in a single run with high sensitivity and reproducibility. This automated immunoassay system utilizes a microfluidic cartridge pre-loaded with specific capture antibodies for each analyte of interest. Serum samples were thawed, centrifuged to remove any debris, and loaded into the cartridge at the recommended dilution factor to ensure accurate detection within the dynamic range of the assay. Each sample was tested in triplicate. The Simple Plex system automatically handled all pipetting, washing, and incubation steps. Following the immunoassay, the cartridge was analysed by the ELLA reader, which measured the fluorescence intensity of the detection antibodies bound to each analyte. The results were automatically generated as analyte concentrations (pg/mL) using built-in software that calculates values based on pre-defined standard curves. The use of ELLA minimized variability and reduced processing time compared to traditional ELISA methods, making it an ideal tool for high-throughput biomarker analysis. The system's precision and accuracy were verified through quality control checks before each assay run.
