# Supplementary material for: Relationship between Mediterranean diet and periodontal inflammation in a UK population: A cross‐sectional study
Source: J Periodontol. 2025 Sep 15;97(1):85–96. doi: 10.1002/jper.70016 (PMC12902710; doi:10.1002/jper.70016)
Supplement: Supplementary file 3 — Supporting Information [file JPER-97-85-s003.docx]

**Supplementary material 3**

**Multivariate analysis of hs-CRP levels when comparing no periodontitis (healthy + gingivitis) versus mild periodontitis (stages I-II) versus severe periodontitis (stages III-IV)**

|  | *p*-value |
| --- | --- |
| no periodontitis vs mild vs severe periodontitis | 0.272 |
| gender | 0.204 |
| Mediterranean diet adherence | 0.089 |
| Smoking history | <0.001 |
| Ethnicity | 0.009 |
| Cardiovascular disease | <0.001 |
| Diabetes type II | 0.098 |
| Hypertension | 0.508 |
| Rheumatic disease | 0.090 |
| Other medical history | 0.454 |
| Frequency of toothbrushing | 0.003 |
| Interdental cleaning | 0.969 |
| Index of Multiple Deprivation | 0.412 |
| Age | 0.839 |
| BMI | 0.679 |

**Multivariate analysis of IL-6 levels when comparing no periodontitis (healthy + gingivitis) versus mild periodontitis (stages I-II) versus severe periodontitis (stages III-IV)**

|  | *p*-value |
| --- | --- |
| no periodontitis vs mild vs severe periodontitis | <0.001 |
| gender | 0.537 |
| Mediterranean diet adherence | 0.569 |
| Smoking history | 0.758 |
| Ethnicity | 0.897 |
| Cardiovascular disease | 0.810 |
| Diabetes type II | 0.802 |
| Hypertension | 0.551 |
| Rheumatic disease | 0.570 |
| Other medical history | 0.003 |
| Frequency of toothbrushing | 0.343 |
| Interdental cleaning | 0.006 |
| Index of Multiple Deprivation | 0.057 |
| Age | 0.339 |
| BMI | 0.228 |

**Multivariate analysis of IL-6 levels across periodontal stages**

|  | *p*-value |
| --- | --- |
| no periodontitis vs mild vs severe periodontitis | 0.014 |
| gender | 0.275 |
| Mediterranean diet adherence | 0.928 |
| Smoking history | 0.615 |
| Ethnicity | 0.903 |
| Cardiovascular disease | 0.856 |
| Diabetes type II | 0.916 |
| Hypertension | 0.498 |
| Rheumatic disease | 0.555 |
| Other medical history | 0.005 |
| Frequency of toothbrushing | 0.208 |
| Interdental cleaning | 0.005 |
| Index of Multiple Deprivation | 0.057 |
| Age | 0.239 |
| BMI | 0.352 |
