# Supplementary material for: Relationship between Mediterranean diet and periodontal inflammation in a UK population: A cross‐sectional study
Source: J Periodontol. 2025 Sep 15;97(1):85–96. doi: 10.1002/jper.70016 (PMC12902710; doi:10.1002/jper.70016)
Supplement: Supplementary file 4 — Supporting Information [file JPER-97-85-s002.docx]

**Supplementary material 4**

**Correlation between adherence to Mediterranean diet and inflammatory biomarkers**

|  | hs-CRP | IL-1b | IL-1a | IL-10 | IL-6 | IL-17 | MMP-8 |
| --- | --- | --- | --- | --- | --- | --- | --- |
| Mediterranean diet adherence score | 0.136 | 0.115 | 0.685 | 0.198 | 0.586 | 0.856 | 0.968 |
